# Impact of vitamin C deficiency on imaging patterns and ventilatory function in pulmonary tuberculosis

**DOI:** 10.3389/fmed.2025.1554723

**Published:** 2025-04-29

**Authors:** Ramona Cioboata, Mara Amalia Balteanu, Ovidiu Mircea Zlatian, Silviu Gabriel Vlasceanu, Mircea Vasile Popescu Driga, Denisa Maria Mitroi, Oana Maria Catana, Cezar Ionut Buciu, Georgiana Camen, Adina Andreea Mirea

**Affiliations:** ^1^Department of Pneumology, University of Medicine and Pharmacy of Craiova, Craiova, Romania; ^2^Department of Pneumology, Victor Babes University Hospital, Craiova, Romania; ^3^Department of Pulmonology, Faculty of Medicine, Titu Maiorescu University, Bucharest, Romania; ^4^Department of Microbiology, University of Medicine and Pharmacy of Craiova, Craiova, Romania; ^5^Department of Physiology, “Carol Davila” University of Medicine and Pharmacy, Bucharest, Romania; ^6^Doctoral School, University of Medicine and Pharmacy of Craiova, Craiova, Romania; ^7^Department of Radiology, University of Medicine and Pharmacy of Craiova, Craiova, Romania; ^8^Department of Oral-Dental Prevention, University of Medicine and Pharmacy of Craiova, Craiova, Romania

**Keywords:** pulmonary tuberculosis, spirometry, vitamin C, chest imaging, CT scan

## Abstract

**Background:**

Studies have shown that vitamin C is essential for the immune response to tuberculosis (TB), and that its deficiency may elevate the risk of TB and related complications. This prospective study investigated the association between disease severity, imaging findings and vitamin C levels.

**Methods:**

This study enrolled 109 patients with confirmed pulmonary tuberculosis based on *Mycobacterium tuberculosis* culture. Patients were divided into two groups based on serum vitamin C levels: 59 patients (54.13%) with normal levels and 50 (45.87%) with low levels.

**Results:**

At baseline, patients in the low vitamin C group showed significantly higher bacillar loads, with 86.00% presenting loads of 2+ or higher compared with 59.32% in the normal group (*p* < 0.001). After 2 months of treatment, 83.05% of the normal vitamin C group achieved culture conversion, while only 28.00% of the low vitamin C group reached the same milestone (*p* < 0.001). CT imaging at baseline revealed that the low vitamin C group had a significantly higher mean frequency of the tree-in-bud pattern (2.66 vs. 2.05; *p* < 0.001). Cavitary lesions were more prevalent in the low vitamin C group, in the superior right lobe (0.34 vs. 0.13; *p* = 0.011) and superior left lobe (0.34 vs. 0.14; *p* = 0.012). After 6 months of treatment, the low vitamin C group exhibited a higher prevalence of bronchiectasis (mean involvement in both lungs: 0.58 vs. 0.16; *p* < 0.001), cavitary lesions (0.32 vs. 0.00; *p* = 0.002), and fibrosis (0.90 vs. 0.36; *p* < 0.001). Pulmonary function tests showed greater impairment in the low vitamin C group. The forced expiratory volume decreased by 5.77% compared to 3.59% in the normal group (*p* < 0.001), the forced vital capacity (FVC) decreased by 12.00% vs. 6.67% (*p* < 0.001), and the Tiffeneau index by 3.34 vs. 2.13 (*p* = 0.002). Receiver operating characteristic (ROC) analysis indicated that FVC (AUC = 0.826) and forced expiratory flow (AUC = 0.745) were stronger predictors of treatment success in patients with normal vitamin C levels.

**Conclusion:**

Vitamin C deficiency is correlated with increased disease severity, delayed bacterial clearance, and persistent pulmonary damage in patients with tuberculosis. Vitamin C supplementation can enhance treatment outcomes in tuberculosis therapy.

## 1 Introduction

Tuberculosis (TB) is one of the world’s leading infectious causes of mortality. In 2023, approximately 8.2 million new TB cases were recorded globally, marking an increase from 7.5 million cases in 2022 and 7.1 million in 2019 (1, 2). These figures are significantly higher than the 5.8 million and 6.4 million cases reported in 2020 and 2021, respectively. The observed surge in TB cases in 2022 and 2023 is likely linked to delayed diagnoses and interruptions in treatment caused by healthcare disruptions during the COVID-19 pandemic (1, 2).

The highest TB incidence rates occurred primarily in Southeast Asia and Africa, with Africa alone accounting for approximately 2.55 million cases [95% uncertainty interval (UI): 2.25–2.87 million], corresponding to an incidence rate of 206 cases per 100,000 population. In contrast, European and American regions reported considerably lower incidence rates of 24 and 33 cases per 100,000 population, respectively. High TB-burden countries collectively accounted for around 9.41 million cases (95% UI: 8.62–10.2 million), highlighting a significant disparity between low- and middle-income countries (LMICs) and high-income regions (1, 3, 4).

According to data from 2023, Romania recorded the highest tuberculosis prevalence among countries within the European Union and European Economic Area (EU/EEA), with an incidence rate of 55 cases per 100,000 residents, reflecting an increase from 53 cases in 2022 and 45 cases in 2021. This substantial disparity underscores Romania’s unique challenge regarding TB, positioning it as the European country with the highest TB incidence rate during this period (1, 2, 5, 6).

Considering the essential role of vitamin C in immune regulation and oxidative stress mitigation during TB, we hypothesized that vitamin C deficiency could significantly impact the severity of pulmonary tuberculosis, as measured by bacterial clearance, imaging patterns, and pulmonary function recovery. Despite evidence supporting the immunomodulatory and bactericidal properties of vitamin C (7), data regarding its impact on TB treatment outcomes remain limited. This study aimed to examine how low serum vitamin C levels affect the radiological extent of the disease, pulmonary function parameters, and microbiological response during standard anti-TB therapy.

The World Health Organization aims to eliminate the TB epidemic by 2035 with the objective of reducing TB cases and deaths by 90%–95%. However, progress toward this goal is primarily impeded by the prolonged duration of TB treatment. Adjunctive agents that synergistically enhance the bactericidal action of antibiotics can potentially reduce the duration of conventional treatment and restore the efficacy of antibiotics against resistant strains (8).

Vitamin C plays a critical role in the immune response against tuberculosis, with deficiencies increasing the risk of infection and associated complications. As a potent antioxidant, it enhances anti-tuberculosis drug efficacy by promoting the Fenton reaction, which generates reactive oxygen species (ROS) that induce oxidative damage (9, 10). Mtb is particularly vulnerable to ROS because of its high iron requirement and limited antioxidant defenses. By amplifying Fenton-driven ROS production, vitamin C sterilizes both drug-susceptible and drug-resistant Mtb cultures, causing DNA damage, lipid peroxidation, and redox imbalances. Additionally, vitamin C influences bacterial dormancy by inducing a viable but non-culturable state, which increases drug susceptibility and enhances the efficacy of TB medications, such as pyrazinamide, isoniazid, and rifampin. Beyond its direct antibacterial effects, vitamin C modulates immune responses by reducing apoptosis in macrophages, enhancing neutrophil function, and promoting T cell differentiation and proliferation. These immunomodulatory properties suggest that vitamin C not only impacts bacterial survival but also strengthens the host immune response against TB. By reducing excessive inflammation, promoting macrophage function, and enhancing neutrophil and T cell activity, vitamin C may help regulate immune homeostasis, prevent tissue damage, and improve overall infection control. This makes it a promising adjunct to TB therapy, potentially enhancing treatment outcomes (9, 11, 12).

A study conducted in Medan City demonstrated that pulmonary TB patients receiving vitamin C supplementation alongside ATD exhibited better chest X-ray (CXR) improvements compared to those receiving a placebo, with 52.5% of the supplemented group showing no lesions after 2 months of treatment, compared to 37.5% in the placebo group (10).

Prompt identification and treatment of tuberculosis are essential for its successful management. TB, chest radiography remains the mainstay for the diagnosis of parenchymal disease, whereas computed tomography (CT) is the method of choice for revealing early bronchogenic spread (13, 14).

Chest computed tomography (CT) is an important diagnostic tool for TB (15, 16). CT imaging is essential for the diagnosis and treatment of tuberculosis, particularly when traditional X-rays are inadequate. Recently, artificial intelligence algorithms have been used to analyze CT data, even if the examination by a radiologist is still mandatory (17, 18). For pulmonary tuberculosis, while chest X-rays are commonly used to diagnose parenchymal disease, CT offers superior sensitivity for detecting lymphadenopathy and is the preferred imaging technique for identifying early bronchogenic spread and distinguishing between active and inactive post-primary disease (13).

The combination of vitamin C supplementation and advanced imaging techniques such as CT has significant potential for improving TB management outcomes. These approaches not only enhance diagnostic accuracy but also provide valuable information for tailoring treatment strategies, ultimately contributing to better patient care and disease control.

Although the role of vitamin C in tuberculosis has been established, there is insufficient research examining how vitamin C levels affect TB treatment outcomes, pulmonary function parameters, and radiological extent in patients with low vitamin C concentrations (19). To the best of our knowledge, no study in Romania has investigated the influence of vitamin C levels on the progression of anti-TB treatment.

By examining the relationship between vitamin C deficiency and the timely evolution of AFB loads in sputum, both through microscopy and culture methods, we aimed to elucidate whether the vitamin C status influences bacterial clearance during treatment. This investigation provides valuable insights into the potential role of vitamin C supplementation as an adjunct to standard TB therapy.

## 2 Materials and methods

### 2.1 Study design and population

A prospective investigation was conducted to examine the association between disease severity, extent of imaging lesions, and serum vitamin C levels in patients with confirmed pulmonary tuberculosis.

This study included 109 patients with confirmed pulmonary tuberculosis who were admitted to the Department of Pneumology at Victor Babes University Hospital in Craiova, Romania between January 2023 and December 2024.

The study adhered to ethical standards, including voluntary participation with informed consent and the principles of the Declaration of Helsinki. Ethical approval was obtained from the University of Medicine and Pharmacy of Craiova (no. 204/05.08.2024) and the Victor Babes University Hospital (no. 24616/17.06.2022).

Study participants were enrolled following their voluntary informed consent, without any form of political, social, or religious discrimination, and in full compliance with data protection legislation.

### 2.2 Inclusion and exclusion criteria

This study investigated the relationship between serum vitamin C levels and pulmonary tuberculosis by selecting a cohort based on stringent inclusion and exclusion criteria.

Initially, a cohort of 235 hospitalized patients with pulmonary tuberculosis was considered, with subsequent focus on the new cases (*n* = 152), as computed tomography (CT) imaging was used to assess the severity of the condition, and previous relapse may have been resolved by pulmonary fibrotic lesions. Tuberculosis (TB) was defined as the presence of at least one sputum specimen yielding a positive acid-fast bacillus test and a positive Xpert MTB RIF test. A new case was characterized as a patient who had not previously received TB medications in combination for a duration exceeding 1 month. All patients included in the study had drug-sensitive tuberculosis and received the standard treatment regimen for 6 months, comprising isoniazid (INH), rifampin (RIF), pyrazinamide (PZA), and ethambutol (EMB) administered daily for 2 months, followed by isoniazid and rifampin for the subsequent 4 months in accordance with the National Tuberculosis Control Program guidelines from Romania. We excluded 43 patients based on the following predefined criteria: absence of bacteriological confirmation (*n* = 11), patients lost to follow-up (*n* = 16), HIV-positive status (*n* = 2), presence of other significant comorbidities (*n* = 10), and gastrointestinal disorders potentially affecting vitamin C absorption (*n* = 4). The study excluded individuals with significant comorbidities, as these conditions could independently affect immune function, nutritional status, and overall disease severity, thereby complicating the accurate assessment of the role of vitamin C in tuberculosis outcomes. Specifically, subjects with chronic respiratory diseases, such as interstitial lung disease, pulmonary fibrosis, bronchiectasis, asthma, chronic obstructive pulmonary disease, or cancer, were excluded. Additionally, individuals with gastrointestinal disorders known to impair vitamin C absorption, such as inflammatory bowel disease, celiac disease, chronic pancreatitis, and extensive gastrointestinal surgery, were excluded. The rationale for excluding patients with gastrointestinal disorders that might impair vitamin C absorption was that including these patients could have led to the misclassification of vitamin C deficiency, as low levels might have been caused by increased metabolic demand associated with tuberculosis. Furthermore, gastrointestinal disorders often result in generalized malnutrition, independently affecting immune function, disease severity, and response to TB treatment, thereby introducing additional bias and confounding variables. Individuals with a history of pulmonary tuberculosis or those treated for latent TB infection were also excluded.

**FIGURE 1:**
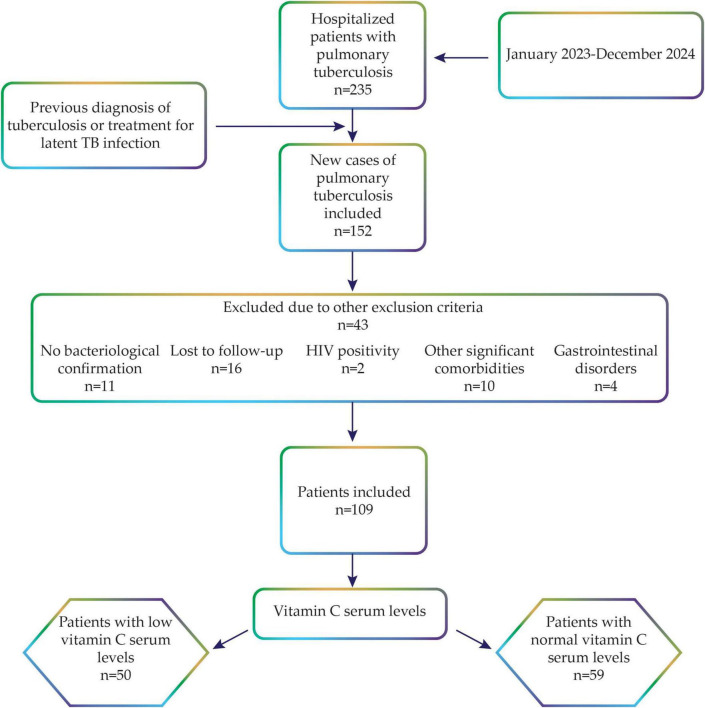
Criteria used to determine the final cohort of study participants.

Additionally, HIV-positive patients and those lacking comprehensive medical records or essential study information were excluded from the study. Lastly, participants receiving treatments that could significantly affect serum vitamin C levels, including antipsychotic medications or chemotherapy, were excluded from the study. After applying these criteria, 109 eligible patients were included in the final analysis (Figure 1).

### 2.3 Follow-up

In accordance with the National Tuberculosis Control Program guidelines from Romania, subsequent evaluations were conducted 2, 4, and 6 months post-initial diagnosis. The 6 months follow-up duration was chosen because it aligns with the standard treatment regimen for drug-sensitive tuberculosis, allowing comprehensive monitoring of patient response and treatment efficacy over the entire course of therapy.

These assessments aim to monitor patient progression and identify alterations in health status throughout the treatment period.

Patient adherence to tuberculosis treatment was rigorously monitored throughout the study. During the first 2 months, all patients were hospitalized, and anti-TB medication was directly administered daily by the nursing staff to ensure strict adherence.

Following hospital discharge, patients continued their treatment in an ambulatory setting, with adherence closely monitored through weekly follow-up visits, involving structured patient interviews to verify medication intake and promptly address any adherence-related issues. Patients were explicitly instructed to refrain from using over-the-counter vitamin supplements throughout the study, and compliance with these nutritional guidelines was verified monthly via structured patient interviews.

All evaluations were conducted in ambulatory settings as per the National Program, ensuring accessibility and consistency in patient care. Evaluations included repeated bacteriological and imaging assessments, enabling a comparative analysis across different time points. Specifically, CT scans and spirometry were performed at baseline and after 6 months of treatment. CT data were independently reviewed by two radiologists. Regular reminders and ongoing support were implemented to encourage patient participation in all scheduled evaluations, and any patients lost to follow-up or with incomplete data were excluded from the final analysis to maintain the integrity and reliability of the findings.

### 2.4 Data collection

Upon admission, a comprehensive health history and physical examination were performed. The findings of the medical evaluations were obtained from patients’ electronic health records.

### 2.5 Laboratory diagnosis of tuberculosis

Sputum collection was performed under standardized conditions to ensure sample quality and reliability. Each subject provided three early morning sputum specimens for analysis. Two distinct identification methods were used in this study. Acid-fast bacilli (AFB) sputum examination was conducted microscopically, with analyses performed initially and at 2, 4, and 6 months post-treatment initiation. Pulmonary tuberculosis was diagnosed using the application of the Ziehl-Neelsen staining technique. This method involves the detection of acid-fast bacilli (AFB) through microscopic examination using light-bright-field microscopy. Lowenstein-Jensen culture was utilized for the confirmation of tuberculosis through the cultivation of *Mycobacterium tuberculosis*, as it remains the gold standard for tuberculosis confirmation. Additionally, a nucleic acid amplification test (GeneXpert, Cepheid, Sunnyvale, CA, United States) using the Xpert MTB RIF and Xpert MTB/RIF Ultra kits was used to detect the *Mycobacterium tuberculosis* complex and mutations associated with resistance to rifampicin (RMP), isoniazid (INH), fluoroquinolones (FLQ), and ethionamide (ETH).

The classification of sputum smear microscopy and culture grading was determined using the following criteria: a positive-1–9 AFB rating was assigned when fewer than 30 colonies were observed; a positive 1+ rating was designated for 30–100 colonies; a positive 2+ rating was applied when more than 100 distinct colonies were present, and a positive 3+ rating was employed for uncountable confluent colonies.

### 2.6 Measurement of serum levels of vitamin C

To ensure consistent baseline measurements, fasting blood samples (6 mL venous blood) were collected in EDTA tubes. To preserve the stability of vitamin C and minimize oxidation and degradation, the samples were handled under controlled conditions. Immediately after collection, the sample tubes were placed in ice water and transported to the hospital laboratory within 30 min. Upon arrival, the samples were centrifuged at 1,000 × *g* for 15 min at 8°C. Plasma was separated, aliquoted, and stored at −*80*°C to prevent vitamin C degradation (20).

During processing, the samples were kept cool, and exposure to direct light was avoided because of the sensitivity of vitamin C to oxidation. Special care was taken to prevent hemolysis because the release of intracellular ascorbate from red blood cells can artificially increase plasma vitamin C levels. This standardized protocol was implemented to ensure the accuracy of vitamin C measurements (20).

Serum vitamin C levels were analyzed using the enzyme-linked immunosorbent assay (ELISA) method with a vitamin C (ascorbic acid) ELISA Kit (Abbexxa, Cambridge, United Kingdom), following the manufacturer’s instructions. Final measurements were obtained using a ChroMate 4300 Plate Reader (Awareness Technology, Palm City, CA, United States).

Serum vitamin C levels were classified according to the manufacturer’s guidelines for ELISA kits. Specifically, values below 0.6 mg/dL were defined as “low vitamin C levels” or “vitamin C deficiency,” whereas levels ranging from 0.6 to 2 mg/dL were considered “normal vitamin C levels” (21).

### 2.7 Pulmonary function test—spirometry

All enrolled patients underwent spirometry using the same SpiroLab IV apparatus (Medical International Research Company, Roma, Italy). To minimize the risk of contamination, single-use turbines and mouthpieces were used for each participant. Throughout all the evaluation procedures, a single spirometer was used in accordance with the optimal operating conditions specified by the manufacturer. A single operator conducted all the assessments. For the proper execution of the test and interpretation of the results, adherence to the American Thoracic Society (ATS)/European Respiratory Society (ERS) guidelines for spirometry standardization was maintained. The assessment of respiratory function complied with the criteria delineated by international standards. We ensured that the patients did not smoke in the hours prior to lung testing. Patients were instructed to abstain from smoking during the hours preceding pulmonary function testing. The absolute values and percentage of predicted values for forced expiratory volume in one second (FEV1), forced vital capacity (FVC), and forced mid-expiratory flow (FEF 25%–75%) were recorded. Furthermore, the FEV1/FVC ratio was calculated for each individual patient (22).

### 2.8 Statistical analysis

Statistical analysis was performed using appropriate statistical tests to determine the significance of the changes in AFB load in sputum and computed tomography scan results before and after tuberculosis treatment in patients with low vitamin C levels. All statistical analyses were performed using the STATA software (StataCorp Ltd., College Station, TX, United States). The sample size calculation revealed that to identify a 40% disparity in TB conversion rates between patients exhibiting normal (estimated at 30%) and low (estimated at 70%) vitamin C levels, utilizing a significance level of 0.05 and a statistical power of 80%, a cohort of 21 patients per group was necessary.

All percentages were calculated based on the total number of patients in each group at each timepoint. Statistical comparisons were performed using the chi-squared test for categorical variables. Differences in continuous variables between groups were analyzed using Student’s *t*-test.

A multivariate random-effects regression model was used for the longitudinal data analysis, which is particularly effective for analyzing repeated measurements from the same subjects over time, accounting for intra-individual correlations that are inherent in longitudinal studies. In the multivariate random-effects regression model, we controlled for potential confounding variables that may influence sputum AFB load, including gender, smoking status, and underweight status. These variables were selected based on previous literature, suggesting an association with tuberculosis disease severity and treatment outcomes. Gender and smoking have known immunomodulatory effects, while being underweight is a marker of malnutrition, which can influence both vitamin C levels and TB progression. Including these covariates helped to isolate the specific contribution of serum vitamin C levels to changes in AFB load over time.

A *p*-value of less than 0.05 was considered indicative of statistical significance.

## 3 Results

The study involved 109 participants, comprising 31 females and 78 males, who were divided into two groups based on their vitamin C levels (Table 1). All patients were confirmed to have drug-sensitive tuberculosis by culture and polymerase chain reaction (PCR) testing for *Mycobacterium tuberculosis* DNA. All participants received standard treatment for drug-sensitive tuberculosis with weight-based dosing of INH at 5 mg/kg body weight, RMP at 10 mg/kg body weight, PZA at 25 mg/kg body weight, and EMB at 15 mg/kg body weight daily for 2 months. For the next 4 months, they received daily INH at 5 mg/kg body weight and RMP at 10 mg/kg body weight.

**TABLE 1:** Demographic parameters of the patients stratified according to serum levels of vitamin C.

Parameter	Normal vit. C (*n* = 59)	Low vit. C (*n* = 50)	Total (*n* = 109)
**Gender^NS^**			
Males	39 (66.10%)	39 (78.00%)	78 (71.56%)
Females	20 (33.90%)	11 (22.00%)	31 (28.44%)
Age (years)^NS^	44.94 ± 15.43	45.81 ± 12.53	45.41 ± 14.12
**Age group^NS^**			
18–40 years	20 (33.90%)	19 (38.00%)	39 (35.78%)
41–60 years	26 (44.07%)	24 (48.00%)	50 (45.87%)
> 60 years	13 (22.03%)	7 (14.00%)	20 (18.35%)
BMI^NS^	19.93 ± 2.33	19.61 ± 2.11	19.79 ± 2.23
**Smoking^NS^**			
Smokers	31 (52.54%)	31 (62.00%)	62 (56.88%)
Non-smokers	28 (47.46%)	19 (38.00%)	47 (43.12%)

NS, non significant *p*-value (> 0.05); BMI, body mass index.

The distribution of individuals was nearly even between those with normal and low vitamin C levels, with 59 participants (54.13%) classified as having normal vitamin C levels and 50 (45.87%) classified as having low levels. The variability in vitamin C levels was notable across the cohort, ranging from 0.41 to 2.32 mg/dL, with a mean value of 0.90 mg/dL and a standard deviation of 0.51 mg/dL, indicative of considerable variation within the sample. The classification into low and normal vitamin C groups followed the manufacturer’s instructions for the ELISA kit, which defines the normal range for serum vitamin C levels as 0.6–2 mg/dL. Levels below 0.6 mg/dL were considered low.

This study highlighted the significant predominance of male participants, accounting for 71.56% of the total sample. The average age of individuals with low vitamin C levels was 44.94 years, slightly lower than the 45.81 years observed in those with normal vitamin C levels. Age distribution analysis revealed a substantial representation of participants aged 41–60 years. Participants in both groups maintained a normal body mass index (BMI), although those with normal vitamin C levels had a marginally higher mean BMI. Additionally, 62.00% of individuals with low vitamin C levels were identified as smokers, suggesting a potential link between smoking habits and vitamin C deficiency.

### 3.1 Analysis of microscopy and culture AFB load

#### 3.1.1 Univariate analysis

At baseline, significant differences were observed in the acid-fast bacilli (AFB) load between the two groups.

At the initiation of treatment, sputum smear microscopy revealed a higher bacterial burden in patients with low vitamin C levels than in those with normal vitamin C levels. In the low vitamin C group, 86.00% (43/50) of the patients had an AFB load of 2+ or higher, with 38.00% (19/50) exhibiting 2+ and 48.00% (24/50) showing 3+ loads. Only 14.00% (7/50) of patients had an AFB count of 1+. In contrast, the normal vitamin C group had a lower proportion of high AFB loads (*p* < 0.001): 59.32% (35/59) had an AFB load of 2+ or higher, and 6.78% (4/59) were AFB-negative at baseline.

**FIGURE 2:**
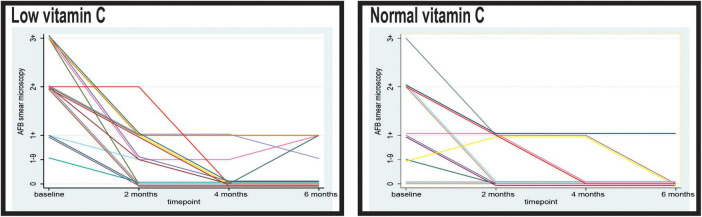
Timely evolution of the microscopy acid-fast bacilli (AFB) load in sputum of the patients with low and normal levels of vitamin C. AFB smear microscopy stands for microscopical Mtb load with semiquantitative assessment from 0 to 3+.

In the second month of observation, a higher proportion of patients with normal vitamin C levels demonstrated negative sputum smear results than those with low levels (Figure 2). In the normal vitamin C group, 50.85% (30/59) of the patients were AFB-negative, whereas only 38.00% (19/50) of the patients in the low vitamin C group were AFB-negative (*p* = 0.079). Furthermore, the low vitamin C group exhibited higher residual bacterial loads, with 4.00% (2/50) still presenting an AFB load of 2+ and 42.00% (21/50) with 1+, compared with 45.76% (27/59) with 1+ in the normal vitamin C group (*p* = 0.653).

At 4 months, the majority of patients in both groups demonstrated negative sputum smear results; however, the low vitamin C group exhibited a slight delay in conversion. In the normal vitamin C group, 84.75% (50/59) were AFB-negative compared with 80.00% (40/50) in the low vitamin C group. Persistent low-level positivity (AFB 1–9 and 1+) was observed in 20.00% (10/50) of the low vitamin C group, whereas 15.25% (9/59) of the normal vitamin C group remained smear-positive at the 1+ level (*p* = 0.257).

At 6 months, 93.22% (55/59) of the patients in the normal vitamin C group achieved smear negativity, compared with only 82.00% (41/50) in the low vitamin C group (*p* = 0.964).

Regarding the culture results, in the low vitamin C group, a higher proportion of patients exhibited elevated AFB loads, with 54.00% (27/50) presenting an AFB load of 2+ and 24.00% (12/50) with 3+. Conversely, in the normal vitamin C group, 47.46% (28/59) of the patients had an AFB load of 1+ and only three had an AFB load of 3+ (*p* = 0.002).

**FIGURE 3:**
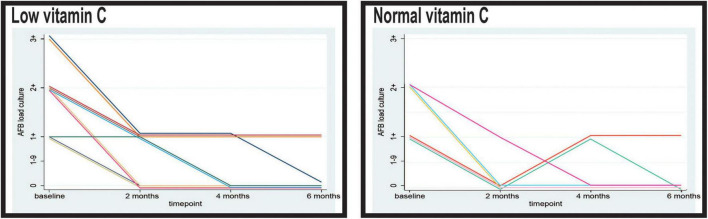
Timely evolution of the culture acid-fast bacilli (AFB) load in sputum of the patients with low and normal levels of vitamin C. AFB smear microscopy stands for microscopical Mtb load with semiquantitative assessment from 0 to 3+.

After 2 months of anti-tuberculosis therapy, a stark contrast in sputum culture conversion rates was evident between the two groups. Most patients (83.05%) in the normal vitamin C group achieved negative sputum cultures, indicating complete microbiological remission at this early stage (Figure 3). In contrast, only 28.00% (14/50) of the patients with low vitamin C levels converted to negative sputum cultures at 2 months, while the majority (70.00%, 35/50) continued to exhibit an AFB load of 1+. The difference in sputum conversion rates at this time point between the two groups was statistically significant (*p* < 0.001).

By the fourth month, the proportion of patients with negative sputum cultures had increased in both groups. In the low vitamin C group, 58.00% (29/50) of the patients achieved sputum culture negativity, whereas 42.00% (21/50) still demonstrated an AFB load of 1+. In the normal vitamin C group, 84.75% (50/59) of patients maintained negative sputum cultures, with only 15.25% (9/59) showing a persistent AFB load of 1+. The difference in sputum conversion rates remained significant at this stage (*p* = 0.002).

Following completion of the 6 months treatment protocol, 53 of the 59 patients (89.83%) in the group with normal vitamin C levels demonstrated consistently negative sputum cultures. Only six patients (10.17%) in this group had an AFB burden of 1+. In comparison, the group with low vitamin C levels in 34 out of 50 patients (68.00%) achieved sputum culture negativity, while 16 patients (32.00%) continued to exhibit an AFB load of 1+. The observed disparity in sputum conversion rates at the end of treatment was statistically significant (*p* = 0.005).

#### 3.1.2 Multivariate analysis

The multivariate analysis used a random-effects longitudinal regression model to analyze the timely evolution of microscopic AFB load in sputum over 6 months of anti-tuberculous treatment (Table 2). Our multivariate model controlled for gender, smoking, and underweight status, as these factors could independently influence bacterial load or treatment response. Despite adjusting for these confounders, low vitamin C levels remained significantly associated with higher AFB loads by both microscopy and culture, reinforcing its potential role in TB pathophysiology.

**TABLE 2:** Multivariate random-effects model of timely evolution of acid-fast bacilli (AFB) load in sputum evaluated by microscopy and culture over 6 months of anti-tuberculosis treatment.

Risk factors	Coeff. (microAFB) (*p*-value)	Coeff. (cultureAFB) (*p*-value)
Low vitamin C	2.309 (0.010)*	3.278 (< 0.001)*
Gender	2.050 (0.037)*	−0.159 (0.855)
Smoker	−0.323 (0.721)	0.581 (0.122)
Underweight	1.125 (0.217)	1.386 (0.086)
Constant	4.118 (< 0.001)	4.967 (< 0.001)

Coeff., random-effects regression coefficient; microAFB, microscopical AFB load was used as dependent variable; cultureAFB, culture AFB load was used as dependent variable.

*Significant result (*p* < 0.05).

The results indicated that low vitamin C levels were significantly associated with higher AFB load (*p* = 0.010). The positive coefficient aligns with the univariate findings, which suggest that patients with low vitamin C levels tend to have a higher bacterial burden detectable by microscopy. Moreover, the association between gender and microscopic AFB load had a coefficient of 2.050, which was statistically significant (*p* = 0.037). Smoking and being underweight had negligible effects.

Multivariate analysis of sputum culture AFB load during the anti-TB treatment period revealed a significant association with low vitamin C levels (coefficient = 3.278, *p* = 0.001). This finding confirmed the univariate results, emphasizing that patients with low vitamin C levels had substantially higher bacterial loads detectable by culture methods. In contrast, variables such as gender, smoking status, and underweight did not exhibit significant effects on culture AFB load in the multivariate model (*p*-values were all above 0.05). These results suggest that low vitamin C levels are a key factor associated with a greater decrease in sputum AFB load over time, thereby confirming the trends observed in the univariate analysis.

### 3.2 Analysis of CT pulmonary lesions in patients with normal and low vitamin C levels

**FIGURE 4:**
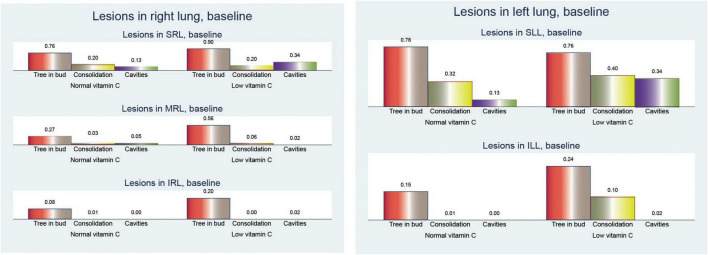
Pulmonary computed tomography (CT) lesions at baseline.

When examining the distribution of CT lesions across different lung lobes at baseline, our study revealed interesting patterns in relation to the vitamin C levels (Figure 4).

At baseline, patients with low vitamin C exhibited a greater mean frequency of the tree-in-bud pattern across both lungs was elevated in the low vitamin C group (2.66 compared to 2.05; *p* < 0.001).

Tree-in-bud opacity was more common in individuals with low vitamin C levels, particularly in the superior right lobe (0.90 versus 0.76; *p* = 0.061), middle right lobe (0.56 versus 0.27; *p* = 0.002), and inferior left lobe (0.20 versus 0.08; *p* = 0.083). However, the superior left lobe displayed a slightly lower occurrence in the low vitamin C group (0.76 versus 0.78; *p* = 0.810).

The differences in consolidation were heterogeneous. In the right lung, consolidation exhibits a slightly higher frequency in the normal vitamin C group (0.34 vs. 0.26; *p* = 0.396). Notably, the superior left lobe showed a higher prevalence of consolidation in the low vitamin C group (0.40 vs 0.32; *p* = 0.402), whereas the superior right lobe had a similar prevalence (0.20 vs 0.21; *p* = 0.965). Conversely, in both lungs, the low vitamin C group demonstrated a higher prevalence (0.76 vs. 0.68; *p* = 0.370).

Cavitary lesions were observed more frequently in patients with low vitamin C levels, particularly in the superior right lobe (0.34 vs 0.13; *p* = 0.011) and the superior left lobe (0.34 vs 0.14; *p* = 0.012). This pattern was consistent across both lungs, with the low vitamin C group presenting a substantially elevated mean (0.72 vs. 0.30; *p* < 0.001).

**FIGURE 5:**
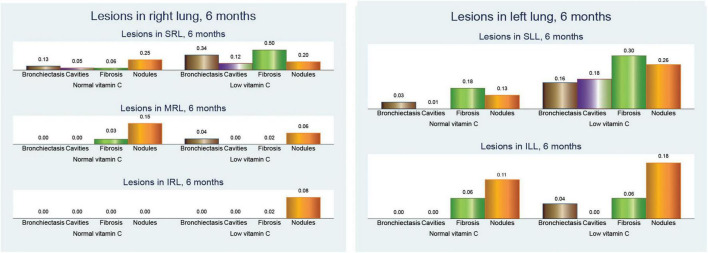
Pulmonary computed tomography (CT) lesions after 6 months of anti-tuberculosis (TB) treatment.

In our analysis of CT findings after 6 months of treatment (Figure 5), we observed notable differences between patients with normal and low vitamin C levels. These findings provide insights into the potential influence of vitamin C status on the resolution of tuberculosis-related lung abnormalities.

Bronchiectasis showed a higher prevalence in the low vitamin C group across all lung regions. In the right lung, bronchiectasis was more frequent in patients with low vitamin C (0.38 vs. 0.14; *p* = 0.003), a pattern that was mirrored in the left lung (0.20 vs. 0.03; *p* = 0.005). When both lungs were considered together, the difference was even more pronounced (0.58 vs. 0.16; *p* < 0.001), suggesting more extensive bronchiectasis in patients with low vitamin C levels.

Cavitary lesions were similarly more prevalent in the low vitamin C group, with mean values of 0.14 in the right lung (*p* = 0.110) and 0.18 in the left lung (*p* = 0.003), compared to no cavities in either lung for those with normal vitamin C levels. This trend was consistent in cavities across both lungs, with a mean increase from no cavities in the normal group to 0.32 in the low vitamin C group (*p* = 0.002).

Fibrosis was also notably more common in the low-vitamin C group. The mean value in the right lung was 0.54 in the low vitamin C group compared with 0.10 in the normal vitamin C group (*p* < 0.001), while that in the left lung fibrosis was 0.36/0.25 (*p* = 0.291), and significantly in both lungs fibrosis was 0.90 in the low vitamin C group and 0.36 in the normal vitamin C group (*p* < 0.001), highlighting a possible link between vitamin C deficiency and fibrotic changes.

Nodular opacities followed a similar pattern, with mean involvement across the right, left, and both lungs showing higher values in the low vitamin C group. Specifically, the mean nodular involvement in both lungs increases from 0.66 in the normal group to 0.78 in the low-vitamin C group (*p* = 0.273).

These results collectively indicate that lower vitamin C levels are associated with more severe and widespread lung changes in TB, even after 6 months of anti-TB treatment, marked by increased bronchiectasis, cavitary lesions, fibrosis, and nodular opacities.

**FIGURE 6:**
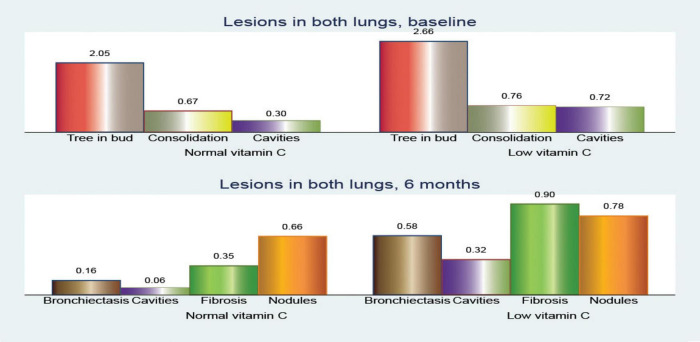
Comparison of pulmonary computed tomography (CT) lesions at baseline and after 6 months of treatment.

Examination of the changes in the total number of cavitary lesions across both lungs (Figure 6) revealed notable differences between individuals with normal and low vitamin C levels. Overall, the reduction in cavitary lesions was more pronounced in those with low vitamin C.

The mean change in the number of cavities showed slight reductions across various lung regions, with no significant differences between the patients with normal and low vitamin C levels. In the superior left lobe, the mean reduction was −0.16 vs. −0.11 (*p* = 0.589). In contrast, the superior and middle right lobes showed modest reductions (mean reduction of −0.22 vs. −0.08; *p* = 0.047 and −0.02 vs. 0.33; *p* = 0.662, respectively), highlighting a smaller overall change in lesion count. However, when considering the number of cavities in both lungs, the reduction was statistically significant (mean reduction, −0.40 vs. 0.23; *p* = 0.069).

This suggests that individuals with low vitamin C levels experience a more substantial decrease in cavitary lesions in both lungs over time; however, we must consider that they also have more severe initial lesion counts.

These findings imply that while low vitamin C levels may be associated with increased lesion severity, there might also be dynamic changes in lesion reduction rates among individuals according to their vitamin C status.

### 3.3 Analysis of pulmonary function parameters in patients with normal and low vitamin C levels

The results provide several insights into the impact of vitamin C levels on lung function, as indicated by the decrease in FEV, FVC, FEF, and Tiffneau index.

#### 3.3.1 Univariate analysis

The mean difference in FEV was higher in the low vitamin C group (5.77) compared to the normal vitamin C group (3.59) (Table 3). This suggests that individuals with low vitamin C levels may have greater changes in their FEV values. The standard deviation was smaller in the low vitamin C group, indicating that these results were more consistent than those in the normal group.

**TABLE 3:** Differences in pulmonary function parameters after 6 months of tuberculosis treatment.

Parameter	Normal vit. C	Low vit. C	P (Kruskal Wallis test)
Difference in FEV (%)	3.59 ± 3.62	5.77 ± 3.04	< 0.001*
Difference in FVC (%)	6.67 ± 4.58	12.00 ± 4.89	< 0.001*
Difference in FEF (%)	11.38 ± 6.39	13.57 ± 6.72	0.087
Difference in Tiffneau	2.13 ± 2.87	3.34 ± 2.17	0.002*

*Significant difference (*p* < 0.05).

The difference in FVC was significantly higher in individuals with low vitamin C levels (12.00) compared to those with normal vitamin C levels (6.67). This indicates that low vitamin C levels may be associated with greater changes in lung capacity. The standard deviation in the low vitamin C group was also higher, indicating greater variability in the response.

The change in FEF was higher in the low vitamin C group (13.57) compared to the normal vitamin C group (11.38). Although the difference is not as pronounced as that with FVC, it still suggests that low vitamin C levels might be associated with larger changes in expiratory flow. Both the groups showed comparable levels of variability.

Forced vital capacity ratio (Tiffneau index) also showed a greater change in the low vitamin C group (3.34) compared to the normal group (2.13). This implies that individuals with low vitamin C levels might have a greater impact on airway function, which could be related to higher airway resistance or obstructive changes.

#### 3.3.2 ROC analysis

**FIGURE 7:**
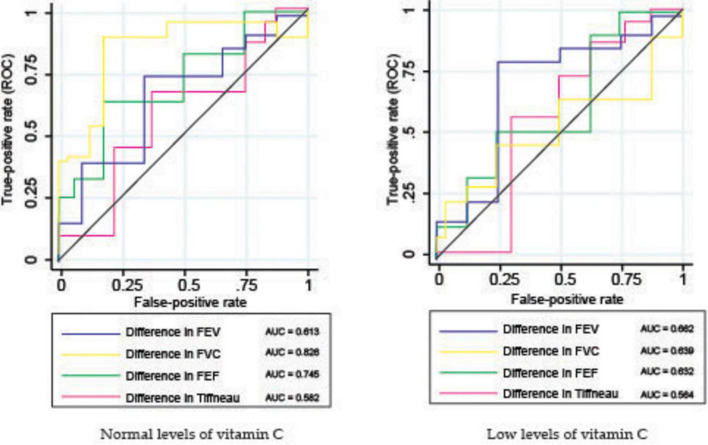
Receiver operating characteristic (ROC) analysis of pulmonary function parameters.

In patients with normal vitamin C levels, the ROC curves showed a moderate level of variability among the parameters (Figure 7 and Table 4), indicating that FVC and FEF are better predictors of TB culture negativity after treatment than FEV1 and TI. The AUC values likely range between 0.6 and 0.8, suggesting moderate predictive power.

**TABLE 4:** Receiver operating characteristic (ROC) analysis (AUC values) of pulmonary function parameters.

Parameter	Normal vit. C	Low vit. C
Difference in FEV	0.613	0.662
Difference in FVC	0.826	0.639
Difference in FEF	0.745	0.632
Difference in Tiffneau	0.582	0.564

In contrast, for patients with low vitamin C levels, the ROC curves were less varied and exhibited different characteristics. ROC curves for FEF and FVC showed better classification, whereas FEV1 appeared to have low predictive power.

## 4 Discussion

This study aimed to investigate the changes in CT during tuberculosis treatment in patients with low vitamin C levels. These results provide valuable insights into the relationship between vitamin C levels and the clinical course of pulmonary tuberculosis. The vitamin C levels of the participants were distributed fairly evenly between the low and normal groups, which helped ensure that the study results were not biased by the uneven distribution of vitamin C levels. The sample size of 109 patients was relatively large, suggesting that the findings are likely generalizable to other populations.

To ensure the accuracy of the vitamin C measurements, a standardized collection and processing protocol was followed to minimize degradation and prevent potential confounders. Fasting blood samples were transported in ice water and processed within 30 min to prevent oxidation. Plasma was separated, aliquoted, and stored at −*80*°C. Careful handling minimized hemolysis and prevented artificial increase in vitamin C levels. These measures helped to ensure the reliability of our vitamin C data and strengthened the validity of our findings. The absence of reported outliers or inconsistencies in vitamin C levels across participants suggests that hemolysis was not a major confounding factor in this study.

This study highlights a significant imbalance in the gender distribution of participants, with males accounting for 71.56% of the sample. While this might reflect the population prevalence of tuberculosis, it is important to acknowledge that this imbalance might limit the generalizability of the findings to a broader population (23, 24).

Although there was a higher percentage of males in the low vitamin C group, the difference was not statistically significant (*p* > 0.05). This male predominance aligns with the global TB epidemiology, where males are generally more affected than females. Worldwide, males consistently exhibit higher tuberculosis (TB) incidence and prevalence rates, likely due to biological and social factors, including differences in immune response and exposure to TB risks, such as smoking and occupational hazards (25–27). Furthermore, males generally have lower vitamin C levels, partly due to their larger fat-free mass and different dietary habits, which could explain the higher proportion of males in the low vitamin C group (28, 29).

The lack of a significant difference suggests that gender is unlikely to confound the relationship between vitamin C levels and TB severity in this cohort, consistent with other studies that also found no strong evidence for gender-based confounding in similar analyses (30).

The marginally elevated mean age of subjects with low vitamin C levels (45.81 years) compared to those with normal levels (44.94 years) indicates a potential association between age and vitamin C deficiency, with older individuals, particularly those in institutional care (31) or with inadequate dietary intake (32) exhibiting increased susceptibility to reduced vitamin C levels and associated health complications.

Elderly populations exhibit lower circulating vitamin C concentrations despite similar intake compared to younger individuals, particularly at intakes below 75 mg/day (32). The elevated prevalence of vitamin C deficiency in patients with tuberculosis aged 18–40 years can be attributed to the impact of the disease on the body’s vitamin levels, increased metabolic demands, and inadequate nutritional intake and absorption (33). The observation that participants across both groups maintained a normal BMI, but those with normal vitamin C levels exhibited a marginally higher mean BMI, suggests that the relationship between vitamin C deficiency and BMI is complex. Multiple studies have demonstrated an inverse correlation between plasma vitamin C level and BMI. Individuals classified as overweight or obese exhibited a higher prevalence of vitamin C deficiency than those with a normal weight. This deficiency was more pronounced in women (34). Vitamin C concentrations exhibit an inverse correlation with adiposity indicators such as waist circumference and body fat percentage (35). Furthermore, reduced plasma vitamin C concentrations are observed in individuals with prediabetes and type 2 diabetes mellitus (T2DM), a condition frequently associated with elevated BMI (36).

The most significant finding was that 62.00% of individuals with low vitamin C levels were smokers. This observation suggests a potential association between smoking and vitamin C deficiency. This finding is consistent with previous research demonstrating that smoking can deplete vitamin C levels (37). Further research is needed to explore the causal relationship between smoking and vitamin C deficiency, and its impact on tuberculosis.

Patients with low vitamin C levels exhibited a higher initial bacterial burden than those with normal vitamin C levels. This was evidenced by the higher proportion of patients with AFB loads of 2+ or higher in the low vitamin C group (86.00% vs. 59.32%). This finding suggests that vitamin C deficiency may be associated with a more severe initial disease presentation, consistent with other studies (38).

The delayed sputum smear conversion observed in the low vitamin C group suggests a potential role for vitamin C in enhancing the early bactericidal activity of anti-tuberculosis medications (10, 39).

The observed delay in sputum smear and culture conversion in the low vitamin C group could be influenced by additional nutritional deficiencies and comorbidities (40–47). Tuberculosis is often associated with malnutrition, which can impair the immune function and delay bacterial clearance. Deficiencies in other micronutrients, such as vitamin D, zinc, and iron, have been linked to impaired immune responses and prolonged TB infection. Vitamin D plays a critical role in macrophage activation against *Mycobacterium tuberculosis*, and low levels have been associated with delayed sputum conversion (24). Zinc deficiency impairs T cell function and macrophage activity, further exacerbating disease severity. Iron status can also influence TB progression, as both iron deficiency and overload affect the immune response. However, *Mycobacterium tuberculosis* has developed mechanisms to maintain iron homeostasis even under immune-imposed iron limitation, allowing it to persist within the host despite low iron levels (42).

Human immunodeficiency virus infection can influence the levels of certain vitamins in patients, including those with TB. Specifically, deficiencies in vitamins D and A have been associated with an increased risk and progression of TB in HIV-infected individuals (45). Multiple studies indicate that TB patients with diabetes have lower serum vitamin D levels compared to those without diabetes, with the deficiency being more pronounced in patients with uncontrolled diabetes and those with a longer history of the disease. Additionally, gastrointestinal (GI) disorders can lead to generalized malnutrition, affecting multiple micronutrients such as iron, vitamin D, and B vitamins, which in turn may influence immune function and TB severity (48–50).

To minimize confounding, we excluded patients with significant comorbidities that could impact TB progression and treatment response, including chronic respiratory diseases, malignancies, GI disorders affecting nutrient absorption, and conditions such as HIV and uncontrolled diabetes. However, despite these exclusions, it is still possible that subclinical nutritional deficiencies or undiagnosed conditions influenced disease severity and response to treatment. Another important consideration is the higher baseline bacillary load observed in the low vitamin C group, which could inherently contribute to delayed bacterial clearance. To address this, we controlled for the initial bacterial load in our statistical analysis by using longitudinal regression in the multivariate analysis section.

Furthermore, strict protocols were followed to ensure the accuracy of vitamin C measurements, including immediate processing and storage under controlled conditions to prevent degradation. The absence of reported outliers or inconsistencies in vitamin C levels across participants suggests that hemolysis was not a major confounding factor in this study.

The rate of sputum smear conversion was lower in the low vitamin C group. At 2 months, 50.85% of patients with normal vitamin C levels achieved smear negativity compared to only 38.00% in the low vitamin C group. This trend persisted for 4 months, albeit with a smaller difference. Delayed smear conversion in the low vitamin C group suggests that vitamin C may play a role in the early bactericidal activity of anti-TB drugs, and highlights the potential significance of vitamin C levels in tuberculosis treatment outcomes (10, 51).

The difference in the culture conversion rates was even more pronounced. At 2 months, 83.05% of patients with normal vitamin C levels achieved culture negativity compared to only 28.00% in the low vitamin C group (*p* < 0.001). This significant disparity persisted throughout the treatment course, with 89.83% vs. 68.00% achieving culture-negativity at 6 months (*p* = 0.005).

Multivariate analysis confirmed the univariate results, highlighting that patients with low vitamin C levels had a substantially worse response to anti-tuberculosis treatment, as evidenced by higher culture AFB loads over time. Variables such as gender, smoking status, and being underweight did not exhibit significant effects, suggesting that vitamin C deficiency is a key factor influencing treatment efficacy. The consistent association between low vitamin C levels and high AFB loads indicates that vitamin C deficiency may hinder bacterial clearance, leading to delayed sputum conversion and prolonged infection.

The observed disparities in culture conversion rates suggest that vitamin C status may significantly influence TB treatment efficacy. Vitamin C is recognized for its antioxidant properties, which potentially mitigate oxidative stress and enhance the immune response against *Mycobacterium tuberculosis*. Through improvement of immune function, vitamin C may facilitate accelerated bacterial clearance, resulting in more rapid culture conversion and improved treatment outcomes.

The observed influence of vitamin C on culture conversion aligns with studies by Xu et al. (33) and Song et al. (12), which have also documented the positive effects of vitamin C supplementation in patients with TB, suggesting it as a potentially beneficial adjunct therapy to standard anti-TB regimens (12, 33).

The increased prevalence of tree-in-bud patterns in the superior lobes of both lungs in the low vitamin C group is consistent with previous observations, suggesting that low vitamin C may be associated with more severe and extensive pulmonary manifestations in tuberculosis (52). Statistical analysis identified low vitamin C levels as a significant risk factor for increased bronchiectasis, cavitary lesions, and fibrosis. Although disease severity may contribute to these findings, it is likely that patients with lower vitamin C levels experience more severe disease progression, which could mediate these outcomes. This suggests that vitamin C deficiency is not merely a consequence of disease severity but may also play a role in its progression.

The superior lobes are frequently the primary site of initial infection, and their involvement in tree-in-bud patterns may indicate a more pronounced inflammatory response (53).

Similarly, the finding of a greater prevalence of cavitary lesions in the low vitamin C group aligns with our understanding of the role of vitamin C in immune function. Vitamin C is a potent antioxidant that supports phagocytes, which are essential immune cells responsible for engulfing and destroying *Mycobacterium tuberculosis* (54–56). Cavitary lesions, a hallmark of TB, are frequently observed in TB patients worldwide and are associated with higher bacterial loads, delayed treatment response, and an increased risk of post-TB lung complications (57, 58). In South Africa, a study identified cavitary lesions in 161 patients at baseline, emphasizing their significant presence in areas with a high TB burden. The prevalence of cavitation post-TB treatment varies widely, ranging from 7.4% to 83.7%, depending on study methodology and patient population (54–57, 59).

The heterogeneous distribution of consolidation observed in this study, specifically within the different lobes of the lungs, adds another dimension to the relationship between the vitamin C status and tuberculosis. While the overall prevalence of consolidation might not be drastically different between the low and normal vitamin C groups, the specific lobes affected seem to vary, hinting at potentially distinct mechanisms at play (60).

The slightly higher frequency of consolidation in the left lung of patients with normal vitamin C levels, although statistically insignificant, suggests that sufficient vitamin C might contribute to a slightly more effective control of inflammation in this region. This could potentially result in a more rapid resolution of the inflammatory processes in the right lung of patients with normal vitamin C levels (61).

The higher prevalence of consolidation observed in both lungs of patients with low vitamin C levels could indicate that low vitamin C levels might generally predispose individuals to a more severe inflammatory response, resulting in a higher overall prevalence of consolidation across the entire lung (19, 61).

Notably, patients with low vitamin C levels exhibited a greater mean reduction in cavitary lesions during the treatment period. This finding suggests that while low vitamin C levels may be associated with a more severe initial disease, there may be a more pronounced healing process in these patients during treatment. However, it is essential to emphasize that this observation requires further investigation, particularly because it does not contradict the association between low vitamin C levels with more severe disease at baseline (62).

The observed higher prevalence of bronchiectasis across all lung regions in patients with low vitamin C levels is a matter of concern, as it suggests that vitamin C deficiency may contribute to a heightened inflammatory response within the lungs, leading to more extensive and persistent airway damage (60). Bronchiectasis is a frequent post-TB complication, with prevalence rates ranging from 4.3% to 86%, depending on imaging modalities. In South Asia, particularly in Northern Pakistan, post-TB bronchiectasis is the most common cause of bronchiectasis, with a high frequency of 76% among cases studied (58). The association between vitamin C deficiency and these pulmonary complications highlights its potential role in TB pathogenesis and recovery (60).

The persistence of bronchiectasis, fibrosis, and nodular opacities at 6 months in the low vitamin C group further indicated that vitamin C deficiency may contribute to more extensive pulmonary damage and delayed resolution of lesions. This observation aligns with the finding that the superior lobes, which frequently serve as the primary site of infection, exhibit a higher propensity for these types of lesions (63).

A substantial increase in fibrosis was observed in both lungs, with a mean value rising from 0.35 in normal vitamin C group to 0.90 in the low vitamin C group (*p* = 0.002), indicating a more severe and extensive fibrotic response in patients with low vitamin C levels. This observation suggests that vitamin C deficiency may contribute to more widespread lung damage, potentially resulting in long-term functional impairment and an elevated risk of respiratory complications (64).

While fibrosis was significantly more prevalent in both lungs, the notable increase in fibrosis in the right lung compared to that in the left lung can be attributed to the fact that the right lung is often the primary site of infection. Its greater involvement in fibrosis may reflect a more severe and persistent inflammatory response in this region (19, 61). A systematic review found fibrosis in 25%–70% of post-TB cases, demonstrating its extensive impact on lung function, including restrictive and obstructive spirometry abnormalities (59). Similarly, a study in Nigeria reported fibrotic lesions as the most prevalent radiological finding in 45% of sputum-positive TB patients, suggesting that delayed diagnosis and treatment initiation may contribute to the high burden of lung scarring (65). These findings highlight the urgent need for targeted interventions, including pulmonary rehabilitation and long-term respiratory care, to mitigate the lasting functional limitations associated with fibrosis.

The higher mean involvement of nodular opacities associated with granulomatous inflammation across all lung regions in the low vitamin C group strengthens the association between low vitamin C levels and a more pronounced inflammatory response within the lungs, potentially influencing the overall severity of tuberculosis.

In most patients, the pulmonary function parameters had decreased over the course of the 6 months anti-TB treatment (66). The decline in pulmonary function occurs due to several histopathological abnormalities that develop during TB infection and treatment, such as fibrosis, and bronchiectasis (67). A significant proportion of patients exhibit residual airflow limitation or restrictive patterns even after treatment completion. This is often due to the extent of lung infiltration as part of inflammatory process and damage caused by TB, which does not fully resolve with antimicrobial therapy (68).

Across all pulmonary function parameters, individuals with low vitamin C levels tended to have higher mean differences compared to those with normal vitamin C levels, which could indicate greater sensitivity or vulnerability of lung function in the context of vitamin C deficiency. The variability, as indicated by standard deviation, was generally higher in the low vitamin C group for FVC and the Tiffneau index, suggesting more heterogeneity in how low vitamin C affects different individuals. These findings imply that maintaining adequate vitamin C levels could help stabilize lung function and prevent larger fluctuations, which might be especially important for populations at risk of respiratory issues. However, further research is necessary to establish causation and to understand the underlying mechanisms more clearly.

The ROC curve analysis for patients with tuberculosis (TB) and varying vitamin C levels assessed the predictive power of pulmonary function parameters for TB culture negativity after treatment. The study compared FEV, FVC, FEF, and the Tiffeneau index in patients with normal and low vitamin C levels, showing differences in performance that suggest the role of vitamin C in TB treatment outcomes. Similar studies had observed the improvement of pulmonary function in patients with normal levels of vitamin C as compared with vitamin C deficient patients (39).

For the normal vitamin C group, there is a clear distinction among the different pulmonary metrics, but, on the other hand, in the low vitamin C group, there is less variability among the pulmonary function metrics, and the discriminative power relative to treatment success is less reliable, likely due to weakened immune function and higher oxidative stress (19). The discriminative power of pulmonary function metrics relative to treatment success was more reliable in patients with normal vitamin C levels, as adequate vitamin C levels allowed for a more diverse and pronounced range of recovery outcomes. In the low vitamin C group, the limited improvement potential due to deficiency resulted in less reliable discriminative power, as the metrics did not vary significantly enough to distinguish between different levels of treatment success (39).

The body’s reduced ability to combat infection effectively might decrease the predictability of TB culture-negative conversion by the pulmonary function parameters (69).

Our findings regarding the role of vitamin C deficiency in the progression of pulmonary tuberculosis align closely with previous evidence presented by Patti et al. (19), who reported that vitamin C significantly impacts tuberculosis treatment due to its antioxidant and immunomodulatory properties (19). Similar to our study, Patti et al. (19) highlighted that vitamin C might enhance treatment outcomes through mechanisms such as increased reactive oxygen species (ROS) production, redox imbalance, lipid peroxidation, and subsequent damage to *Mycobacterium tuberculosis* cell walls, thus facilitating bacterial clearance. Furthermore, Patti et al. emphasized the potential synergistic effects of combining vitamin C supplementation with standard anti-TB drugs, potentially enhancing therapeutic efficacy and reducing treatment duration. Our observations of delayed sputum culture conversion and persistent radiological abnormalities in patients with low vitamin C levels provide important clinical validation for the previously suggested mechanistic insights.

In addition, while Xu et al. (33) reported inconclusive findings regarding vitamin C due to limited available studies, they highlighted the critical need for further research on this relationship. Our study directly addresses this gap by providing clear clinical evidence that low vitamin C levels are associated with worse tuberculosis outcomes, including delayed bacteriological clearance and impaired pulmonary function recovery. Moreover, the finding by Patti et al. (19) that high doses of vitamin C exhibit sterilizing effects on drug-resistant *M. tuberculosis* cultures and prevent persistent bacterial populations further reinforces our hypothesis regarding the therapeutic importance of maintaining adequate vitamin C levels in tuberculosis management (19). Taken together, our results and the evidence summarized by Patti et al. (19), Xu et al. (33) indicate significant therapeutic implications for optimizing the vitamin C status in patients with tuberculosis. These combined insights strongly advocate for additional well-designed clinical trials to explore targeted vitamin C supplementation alongside conventional anti-tuberculosis therapies, with the ultimate goal of improving treatment outcomes and reducing disease severity.

Our results align with our recently published preliminary findings from a study conducted in Southwestern Romania (70), where we similarly reported that tuberculosis patients with low serum vitamin C levels exhibited significantly delayed bacteriological clearance after 2 months of treatment, higher inflammatory marker levels, and increased disease severity compared to patients with normal vitamin C levels. The current study expands upon these initial observations by including a larger patient cohort and providing more robust evidence supporting the hypothesis that vitamin C deficiency negatively affects tuberculosis outcomes. These combined findings further strengthen the clinical rationale for evaluating vitamin C supplementation as an adjunct therapy in tuberculosis treatment protocols.

### 4.1 Future directions

Although the findings of this study highlight the potential role of vitamin C in influencing the severity and localization of TB lesions, further research is required. We need to explore how vitamin C deficiency affects different aspects of the immune response, including T cell activation, macrophage function, and cytokine production, in the context of *Mycobacterium tuberculosis* infection.

Furthermore, we need to investigate whether vitamin C supplementation during the course of tuberculosis treatment might improve clinical outcomes, including reducing lesion severity, accelerating sputum conversion, and improving lung function. Conducting extensive clinical trials involving vitamin C supplementation, particularly in patients with documented vitamin C deficiency is essential to determine its potential as a valuable adjunct to standard TB treatment. These trials should also evaluate the optimal dosage, duration, and timing of supplementation to maximize therapeutic benefits while minimizing potential adverse effects. By addressing these research gaps, future studies can provide robust evidence of the clinical utility of vitamin C supplementation in tuberculosis care. This could lead to improved treatment protocols and better patient outcomes, ultimately contributing to global efforts in reducing the incidence and mortality rates associated with TB.

### 4.2 Limitations

This study was conducted at a single hospital in Romania, which limits the generalizability of the findings to other populations and healthcare settings.

This study did not assess dietary intake or overall nutritional status beyond the serum vitamin C levels. Although serum vitamin C serves as a biomarker of vitamin C status, its levels can be influenced by dietary intake, metabolic demand, and absorption efficiency. However, to minimize confounding factors, we excluded individuals with gastrointestinal disorders that could impair vitamin C absorption and those with significant comorbidities affecting metabolism.

However, this study did not examine the effects of vitamin C supplementation on the clinical course of tuberculosis.

The study did not control for potential confounding factors that could influence vitamin C levels and tuberculosis outcomes, such as dietary habits, physical activity, socioeconomic status, and other variables.

The study’s follow-up period of 6 months may be insufficient to fully assess the long-term impact of vitamin C deficiency on the course of tuberculosis. The National TB Program stipulates that if negative at 6 months, treatment is ceased, and the patient is scheduled for a subsequent visit at 12 months from the initiation of treatment.

## 5 Conclusion

This investigation provides compelling evidence suggesting that vitamin C deficiency significantly influences the pathogenesis, progression, and response to treatment of pulmonary tuberculosis. Our findings highlight the potential importance of assessing and addressing vitamin C levels in patients undergoing anti-TB therapy, particularly given the widespread nutritional deficiencies observed in resource-limited settings affected by the global TB epidemic. Further research is essential to investigate the benefits of vitamin C supplementation or dietary interventions aimed at correcting deficiency and potentially improving the effectiveness of standard tuberculosis treatment regimens.

Nevertheless, this study had certain limitations. Owing to its observational design, this study cannot conclusively determine causality. Moreover, sample size might have influenced the statistical power and generalizability of the results. Additionally, confounding factors, such as variability in dietary intake, nutritional status beyond vitamin C, smoking habits, and comorbidities, could influence the observed associations. Therefore, larger-scale, multicenter, randomized controlled trials are necessary to validate these findings, establish causative relationships, and evaluate their applicability across diverse patient populations.

Despite these limitations, our results strongly support the consideration of vitamin C supplementation as a complementary approach to tuberculosis management, with the goal of enhancing patient outcomes and reducing disease severity.

## Data Availability

The original contributions presented in this study are included in this article/supplementary material, further inquiries can be directed to the corresponding authors.
